# Entomopathogenic Action of Wild Fungal Strains against Stored Product Beetle Pests

**DOI:** 10.3390/insects14010091

**Published:** 2023-01-14

**Authors:** Spiridon Mantzoukas, Ioannis Lagogiannis, Foteini Kitsiou, Panagiotis A. Eliopoulos

**Affiliations:** 1 Department of Agriculture, University of Ioannina, Arta Campus, 45100 Ioannina, Greece; 2 ELGO-Demeter, Plant Protection Division of Patras, 26444 Patras, Greece; 3 Laboratory of Plant Physiology, Department of Biology, University of Patras, 26504 Patras, Greece; 4 Laboratory of Plant Health Management, Department of Agrotechnology, University of Thessaly, Geopolis, 41500 Larissa, Greece

**Keywords:** entomopathogenic fungi, stored product pests, *Cladosporium*, *Condenascus*, *Lecanicillium*, *Penicillium*

## Abstract

**Simple Summary:**

Stored product pests cause significant losses to agricultural products every year. Their control depends heavily on the use of fumigants and other insecticides, which have many negative consequences for humans and the environment. Nowadays, the use of fungal entomopathogens is one of the most promising alternatives to reduce the use of chemicals in storage facilities. We tested new wild strains of entomopathogenic fungi from the genera *Cladosporium*, *Condenascus*, *Lecanicillium*, and *Penicillium* in laboratory bioassays on various storage beetles. All strains caused remarkable mortality in adult beetles, reaching 80% in some cases after 21 days. The results of our study show that insect-pathogenic fungi can be effective biological tools for the protection of stored agricultural products. Research to discover new strains with high pathogenicity and to develop new methods for mass production and standardization of entomopathogens should be continued to enable their practical application in the future.

**Abstract:**

There is ample evidence that entomopathogenic fungi can be used as alternative biological control agents for the management of insect pests in storage facilities. As the market demands more environmentally friendly methods and chemical insecticides become increasingly obsolete, more studies are being conducted to evaluate new strains of entomopathogenic fungi for their efficacy in storage facilities. In this context, we tested ten species of fungi isolated from soil, belonging to the genera *Cladosporium*, *Condenascus*, *Lecanicillium*, and *Penicillium*, for their long-term effects on economically important beetle species. Whole wheat was directly sprayed with a conidial suspension of 10^8^ spores/Ml of each of the tested fungi and then adults of *Sitophilus granarius*, *S. oryzae*, *S. zeamais*, *Rhyzopertha dominica*, and *Trogoderma granarium* were placed on the sprayed medium to study the mortality effects. Significantly higher mortality than the control was observed in all treatments. The lowest LT_50_ (9.164 days) was observed in *T. granarium* infected with *Penicillium goetzii*. The isolate with the strongest results was *L. dimorphum*, which recorded remarkably low LT_50_ values in *S. oryzae* (~11 days), *R. dominica* (~12 days), *T. granarium* (~10 days), and *S. granarius* (~13 days). However, for *S. zeamais*, it was more than 16 days. Our results confirm the existing literature on the efficacy of EPF on storage beetles, suggest the possible virulence of wild untested strains, and also highlight the importance of EPF specificity.

## 1. Introduction

In agriculture, financial losses due to pest infestations are not limited to the field but continue into storage. It is estimated that pest infestations of stored products cause annual economic losses of 10% worldwide [1]. Storage pests are mainly beetles and moths [2], which contribute to the spoilage of stored goods not only by feeding on them but also by transmitting harmful microorganisms and contaminating products with their frass and exuviae, which can be harmful to human health [1,3]. Storages offer favorable environmental conditions for the rapid development of these pests, offering ideal temperatures, humidity, and abundant food. Infestation can lead to enormous economic losses as the quality, quantity, and commercial value of stored commodities are affected [4].

Control of these pests is usually based on the use of synthetic insecticides and fumigants, a practice which, despite its effectiveness, is increasingly problematic, as many of these substances are banned due to the health risks associated with their use, as well as the contamination of products with chemical residues that lead to deterioration of nutritional quality and the development of resistance [5]. Recently, over-reliance on phosphine, especially after the restriction of methyl bromide, has already led to increased frequency, prevalence, and severity of resistance in numerous stored-product pests, and the lack of suitable alternatives is worsening these effects day by day [6]. In addition, phosphine can have erosive effects and damage equipment when used repeatedly at high concentrations [7].

Entomopathogenic fungi (EPFs) have been shown to have significant potential to control insects while minimizing the negative effects of insecticides, and they are used in pest management worldwide [8,9,10,11,12]. EPFs exclusively infect insects, the mycelium penetrates the cuticle and grows in the hemocoel, resulting in death, and then sporulation follows on the external surfaces of insects’ cadavers, promoting epizootics [13]. The wide distribution of EPF in a variety of habitats is evidence of its safety, low environmental impact, and low toxicity to mammals [14,15].

Although high mortality rates in various storage insects due to fungal pathogens have been reported in the literature, little attention has been paid to the practical use of such pathogens as biological control agents in storage facilities [16,17,18]. In this regard, three commercially available species have been tested for their potential for protecting stored products from severe pests: *Beauveria bassiana* (Balsamo) Vuillemin (Hypocreales: Cordycipitaceae) [19,20], *Metarhizium anisopliae* (Metschinkoff) Sorokin (Hypocreales: Clavicipitaceae) [21,22], and *Cordyceps fumosorosea* (Wize) (formerly *Isaria fumosorosea*) (Hypocreales: Cordycipitaceae) [23,24,25], In addition to commercially available EPFs, extensive research is being carried out on wild strains collected from nature (from the soil or infected dead insects), isolated in the laboratory and evaluated for their insecticidal ability. This results in the enrichment of our biological «arsenal» for the control of insect pests.

Following this strategy we studied, the long-term efficacy of ten wild fungal isolates of the genera *Cladosporium*, *Condenascus*, *Lecanicillium*, and *Penicillium* isolated from soil in Greece. The effect was determined by measuring the survival time of adults of the granary weevil *Sitophilus granarius*, the rice weevil *S. oryzae*, the maize weevil *S. zeamais* Motsch. (Coleoptera: Curculionoidea), the lesser grain borer *Rhyzopertha dominica* (Coleoptera: Bostrychidae), and the khapra beetle *Trogoderma granarium* Everts (Coleoptera: Dermestidae). The results of our study were analyzed in the context of the intended use of insect pathogens as a key component of integrated pest management in storage facilities. 

## 2. Materials and Methods

### 2.1. Collection, Isolation, and Identification of Fungi

Soil samples were collected in 2019 in Patras, Achaia, Greece. Samples were collected from a depth of 10 cm below the top soil layer and placed in sealed polyethylene bags after excavation. The isolation of the fungal samples was performed according to the bait—methods of Mantzoukas et al. 2019 [25]. The mycelium present on the dead insect baits was inoculated onto a new medium to purify the fungal cultures. The purification of the cultures was performed until the growth of a single colony on Sabouraud Dextrose Agar (SDA) plates was achieved. The morphological characteristics of the strains were observed by inoculating a fungal mycelial plug (1 cm) onto an SDA plate for 10 days. At the end of the growth period, the sporulation structure was taped from the edge with transparent tape and then stained with Phenol cotton blue reagent. Spore morphology was observed under a phase contact microscope (×100) (ZEISS Primo Star, Carl Zeiss Microscopy GmbH, Munich, Germany). DNA sequencing was also performed using the method described by Mantzoukas et al. 2019 [25].

### 2.2. Insects

Mortality bioassays were performed on five important beetle pests: *S. granarius*, *S. oryzae*, *S. zeamais*, *R. dominica*, and *T. granarium*. These species are globally common storage pests that cause severe losses and damage to a variety of commodities. Adults of mixed sexes aged < 2 weeks were collected and transferred to uninfested wheat grains. The adults were left there for 1 week to lay eggs and then removed so that individuals of standardized age could be obtained. All species were reared on durum wheat at 27.5 °C and 75% relative humidity (RH).

### 2.3. Bioassays

Individual batches of 500 g wheat were filled into cylindrical 0.45 L glass jars. The product was sprayed directly with 1 mL of conidial suspension containing 10^8^ spores/mL of the fungus using a Potter spray tower (Burkard Manufacturing Co. Ltd., Rickmansworth, Hertfordshire, UK) at 1 kgf/cm^2^. After the application of EPF, the wheat batches were placed back into the jars and shaken by hand for 30 s to achieve uniform distribution of the fungi. A separate set of batches was sprayed with distilled water only and served as control. Twenty 10 g samples were taken from each jar and placed in a 9 cm Petri dish. The inner “neck” side of each Petri dish was covered with Fluon (Northern Products, Woonsocket, RI, USA) to prevent insect escape. Adults were starved for 24 h. Ten individuals of each beetle species were placed in each Petri dish and then placed in plastic boxes containing saturated sodium chloride solutions to maintain 75% r.h. Petri dishes were then placed in incubators at 27.5 °C and 75% r.h. After 7, 14, and 21 days, all Petri dishes were opened and dead adults were counted. All dead adults were immediately removed and immersed in 95% ethanol for 1 min, washed in sterile distilled water for 5 min, dried, and then placed on moistened filter paper. The above procedure was performed in a laminar flow chamber. The cadavers were kept in the dark at 25 °C for 5–7 days, and those that showed hyphal growth characteristics of entomopathogenic fungi were classified as infected. The fungal species was first identified by microscopic observation based on the shape and size of the hyphal growth and confirmed by PCR analysis. In the present work, the DNA sequences were matched using the Basic Local Alignment Search Tool (NCBI BLAST) [25].

The whole procedure was repeated ten times by preparing new batches of treated and untreated grains for each replicate (10 × 1 × 10 × 5 = 500 Petri dishes for each replicate × dose × fungal strains × insect species).

### 2.4. Data Analysis

All values were arcsine transformed before analysis. Mortality data were subjected to a two-way analysis of variance (ANOVA) to evaluate the main effects and interactions of fungal isolate and exposure time on insect mortality. In the case of significant F values, means were compared using the Bonferroni test. The median lethal time (LT_50_) of tested adults was calculated by probit analysis with a 95% confidence interval (CI). All statistical tests were performed using SPSS (SPSS, Inc., Chicago, IL, USA, version 23).

## 3. Results

The fungal species recovered from the soil and tested for pathogenicity belonged to the genera Cladosporium, Condenascus, Lecanicillium, and Penicillium (Table 1).

Mycelial and conidial growth on cadavers suggested that almost all deaths were due to a pathogen. Observation of the cadavers showed that external mycelium appeared within the first 72 h after they were placed on moist filter paper.

**Figure 1:**
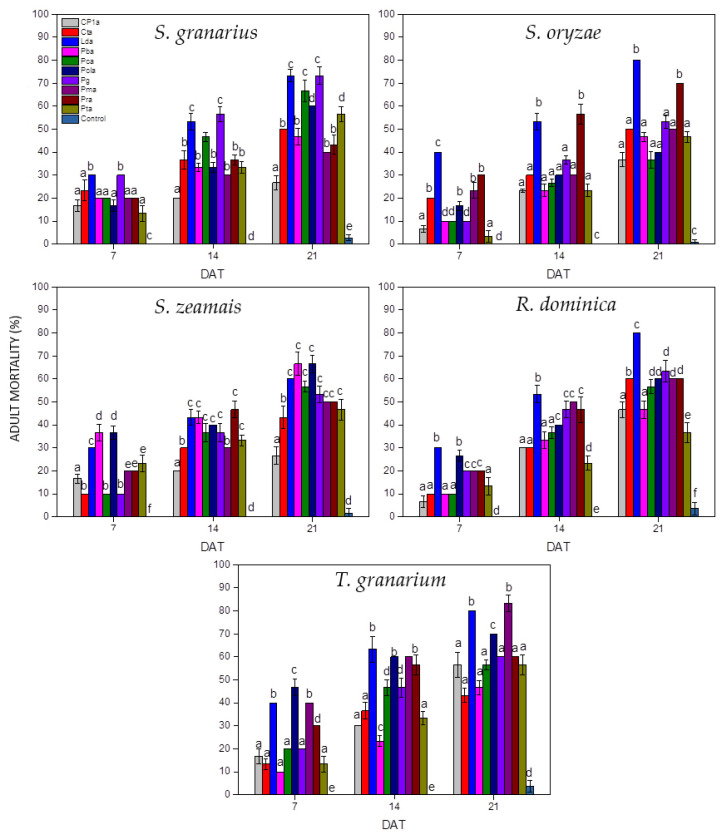
Mean mortalities (%) of experimental adult beetles treated with EPF in the present study (grains were sprayed with conidial suspension at 10^8^ conidia/mL DAT: days after treatment, columns of the same DAT marked with the same letter did not differ significantly, error bars represent standard deviation of the mean).

The average mortality (%) of adult beetles experimentally exposed to EPF in the present study is shown in Figure 1. After 7 days of exposure, the number of dead insects was relatively low (<50%), but then increased significantly and exceeded 80% on day 21 of the experiment (Figure 1).

Probit analysis was used to estimate the median lethal time LT_50_. The probit mortality regression data along with the confidence limits (CL) and other estimated probit parameters are presented in Table 2. These data indicate that *S. zeamais* and *T. granarium* were the most resistant and susceptible to EPF isolates, respectively. However, the differences in LT_50_ among various beetle species and EPF isolates were insignificant (Table 2).

The main effects and interaction of fungal isolate and exposure time on beetle mortality proved to be significant in all cases (*p* < 0.001) (Table 3).

## 4. Discussion

As mentioned earlier, eliminating the use of chemical pesticides and replacing them with alternative methods of pest control in storage is of great importance. The use of fungal strains as biopesticides is not a new approach. Many studies have demonstrated their effectiveness against destructive pests. There is extensive literature that provides results supporting the use of entomopathogenic fungi as a means of integrated pest management in storage facilities [15,16,17,18,19,20,21,22,23,24,25,26,27,28,29,30,31,32,33,34].

Kavallieratos et al. tested the effect of *M. anisopliae* on adult *S. oryzae*, resulting in critical mortality at doses of 1.77 × 10^7^ and 1.77 × 10^8^ conidia/mL [24]. Khashaveh et al. reported that *B. bassiana* can be successfully used to control storage pests in wheat [26]. Wakefield et al. achieved 100% mortality of *Oryzaephilus surinamensis* (L.) (Coleoptera: Silvanidae) after 10 days of treatment with a dose of 10^8^ conidia/mL of some *B. bassiana* isolates [27]. Wakil et al. examined several isolates of *B. bassiana* and *M. anisopliae* against *R. dominica*, *S. granarius*, *T. castaneum*, and *T. granarium*, and found that the first one was the most susceptible, whereas the last one was the most resistant to fungal infection [28]. Finally, Batta treated newly emerged adults *R. dominica* with *M. anisopliae* conidia formulated in wheat flour (86.7%) and inverted emulsion (93.3%) and found high mortality after 7 days [29]. An analytical list with data from relevant lab bioassays is provided by Rumbos and Athanassiou [15].

There have been very few field studies to assess the effectiveness of EPF in storage facilities, despite the abundance of laboratory data that is accumulated from numerous investigations. Stathers provided information from research projects carried out in Africa with contradictory conclusions [30]. In a field trial, stored maize was treated with *B. bassiana* for protection against *P. truncatus*; however, despite the pest densities being substantially lower in treated grains, the losses were significant [31]. *Beauveria bassiana* combined with electrostatic powder was tested as a surface treatment in an empty store, and this produced a sufficient level of control against *O. surinamensis* [32]. Combined application of *B. bassiana* with synthetic insecticides to wheat stored in polypropylene bags provided satisfactory protection for 30 days but after 180 days, grain damage was above acceptable levels despite significantly increased pest mortality [33,34].

For this experiment, ten different fungal species from soil were evaluated for long-term entomopathogenicity in five important storage beetles. All isolates caused significantly higher mortality in treated beetles compared to the control. Most of the species found and tested in this study have not been previously examined against insects.

The genus *Lecanicillium* is known for its entomopathogenic properties and infects a wide range of insects [35,36,37,38,39,40,41,42,43,44]. Strains of *Lecanicillium* (*Verticillium*) *lecanii* Zimmermann are not only commercially available to control severe agricultural pests [36], but have also been shown to be effective against storage pests such as *S. oryzae* [37], *S. zeamais* [38], and the red flour beetle *Tribolium castaneum* Herbst (Coleoptera: Tenebrionidae) [39]. Extracts containing secondary metabolites from *L. attenuatum* exhibited the highest insecticidal activity against the Asian tiger mosquito *Aedes albopictus* (Skuse) (Diptera: Culicidae) and the diamondback moth *Plutella xylostella* (Lepidoptera: Plutellidae) [40]. Larvae of the latter species fed with cabbage leaves sprayed with isolates of *L. muscarium* recorded mortality greater than 80% [41]. The same *Lecanicillium* species caused high mortality in the silverleaf whitefly *Bemisia tabaci* (Gennadius) and the greenhouse whitefly *Trialeurodes vaporariorum* Westwood (Hemiptera: Aleyrodidae) under both controlled laboratory and glasshouse conditions [42,43]. In addition, *L. longisporum* succeeded in controlling populations of the green peach aphid *Myzus persicae* (Sulzer) (Hemiptera: Aphididae [44].

Although many *Lecanicillium* species are known for their insecticidal action, *L. dimorphum*, the species that had the highest mortality in this study, has been poorly evaluated for its entomopathogenic activity in only a few studies. It has been reported to colonize *Phoenicococcus marlatti* Cockerell (Hemiptera: Phoenicococcidae) resulting in 100% parasitism [45], while it caused 56.5% mortality in nymphs of *M. persicae* after 7 days [44]. On the other hand, it caused significant fecundity reduction in the predatory minute pirate bug *Orius laevigatus* (Fieber) (Hemiptera: Anthocoridae) an important and widely distributed biocontrol agent [46]. The present study enriches our knowledge about the potential of this EPF species as the pest control agent.

The genus *Cladosporium* is also known for its insecticidal potential against whiteflies [47], spidermites [48], moths [49], and aphids [47,50], but our report is the first to investigate *C. puyae*, and although its performance was not as successful as other strains, possible virulence to other insects cannot be excluded. The same is true for *Condenascus tortuosus*.

In our study, seven different *Penicillium* species were evaluated for their efficacy against beetles. Some species of the genus *Penicillium* are known for their entomopathogenic activity but most of them are not thoroughly studied. Extracts from *Penicillium* sp. were effective for the control of the tobacco cutworm *Spodoptera litura* (F.) (Lepidoptera: Noctuidae) and the southern house mosquito *Culex quinquefasciatus* Say (Diptera: Culicidae) larvae [51] and caused significant mortality levels in the confused flour beetle *Tribolium confusum* Jacquelin Du Val (Coleoptera: Tenebrionidae) [52]. Moreover, Da Costa et al. investigated the efficacy of *Penicillium* sp. against various mosquitoes, and mortality rates ranged from 0 to 100%, depending on the concentration of conidia [53]. *Penicillium citrinum* and *P. chrysogenum* have also been reported to have insect-pathogenic properties, and *P. chrysogenum* in particular is pathogenic to the African malaria mosquito *Anopheles gambiae* Giles (Diptera: Culicidae) [54]. In a study conducted on *C. quinquefasciatus* larvae, strain *P. citrinum* CM-010 was by far more effective than strains of *M. anisopliae* and *B. bassiana* [55]. In another study, *P. citrinum* CTD-24 caused the highest cumulative mortality in eggs and neonates of the fall armyworm *Spodoptera frugiperda* Smith (Lepidoptera: Noctuidae) [56].

Due to several constraints, including unpredictable environmental conditions, probable chemical residues, the presence of other species, and the implementation of other control measures, the practical application of EPF against storage pests is hindered [27,30]. Basically, the greatest obstacle is the hostile dry environment that makes it difficult for fungi to survive. However, advancements in formulation technology may turn EPF into a useful IPM tool for stored product protection. Additionally, it should be noted that an EPF-based biopesticide may exhibit greater persistence than a chemical pesticide since the entomopathogen may cycle on a dead insect body (cadaver), reintroducing further inoculum into the ecosystem. This internal sporulation may occur even in the dry storage environment [30].

## 5. Conclusions

Apart from the pathogenic activity of the tested fungal species on insects, our study once again confirmed that the efficacy of a given fungal species is always strain-specific and virulence may vary depending on the host. Successful infection and germination depend on both biotic and abiotic factors, such as host susceptibility, host life stage, length of the incubation period, temperature, and humidity [57,58]. From the above literature and our results, it appears that a particular EPF species may be virulent against a particular host while having little to no effect on others. For this reason, extensive screening should be performed to determine the level of virulence acceptable for further development of a fungus-based formulation.

The use of entomopathogenic fungi to control stored-product pests is a promising IPM tool. Despite multiple reports of effective laboratory tests using EPF on storage pests, this has not yet been successfully translated into practice. This would necessitate further development of conidia formulation and a thorough investigation into whether internal sporulation is taking place in infected insects in the store [30]. For EPF to be used as part of an IPM approach, research must be conducted under as realistic field conditions as possible. Knowledge of their effectiveness when paired with other storage IPM strategies (such as insecticides, heat or cooling treatment, other biocontrol agents, inert dust, modified atmospheres, etc.) is also crucial. Apart from that, further experimentation should be conducted to isolate new strains and thoroughly investigate the level of virulence and specificity. Characterization of new species using molecular tools and DNA sequencing can contribute to a better understanding of these organisms by clarifying taxonomic relationships and mechanisms involved in pathogenicity.

## Figures and Tables

**Table 1:** Isolates of several EPF species that were tested in the present study. All collected fungal isolates were lab cultured and stored at 25 °C in SDA plates.

Fungal Species	Isolate Number	Isolate Site (Latitude, Longitude)	Insect Bait
*Cladosporium puyae* Bensch, Crous and U. Braun (Cladosporiales: Cladosporiaceae)	CP1a	Dasyllio 1 (38.248686, 21.747233)	*S. granarius*
*Condenascus tortuosus* (Udagawa and Y. Sugiy.) X. Wei Wang and Houbraken (Sordariales: Chaetomiaceae)	Cta	Dasyllio 8 (38.247116, 21.744431), Elos 4 (38.280985, 21.749813)	*S. granarius*, *T. confusum*
*Lecanicillium dimorphum* (J.D. Chen) Zare and W. Gams (Hypocreales: Cordycipitaceae)	Lda	Dasyllio 3 (38.249872, 21.748449)	*S. granarius*
*Penicillium brevicompactum* Dierckx (Eurotiales: Aspergillaceae)	Pba	Elos 2 (38.281121, 21.747073)	*S. granarius*
*Penicillium chrysogenum* Thom (Eurotiales: Aspergillaceae)	Pca	Elos 3 (38.280382, 21.750789), Dasyllio 9 (38.246973, 21.744602)	*T. confusum*
*Penicillium citrinum Thom* (Eurotiales: Aspergillaceae)	PcIa	Elos 3 (38.280382, 21.750789),	*S. granarius*
*Penicillium goetzii* J.D. Rogers, Frisvad, Houbraken and Samson (Eurotiales: Aspergillaceae)	Pg	Dasyllio 5 (38.249276, 21.746061)	*S. granarius*
*Penicillium murcianum* C. Ramírez and A.T. Martínez (Eurotiales: Aspergillaceae)	Pma	Dasyllio 10 (38.247201, 21.744944)	*T. confusum*
*Penicillium rubefaciens* Quintanilla (Eurotiales: Aspergillaceae)	Pra	Dasyllio 11 (38.246636, 21.744328)	*T. confusum*
*Penicillium thomii* Maire (Eurotiales: Aspergillaceae)	Pta	Dasyllio 4 (38.249504, 21.745850)	*S. granarius*

**Table 2:** Lethal time (LT_50_) and associated probit parameters of tested adults treated with conidial suspension at 10^8^ conidia/mL by several species of entomopathogenic fungi for a period of 7, 14, and 21 days.

Insect Species	Isolate	Slope	Intercept	LT_50_ (95% CL)	χ^2^	R^2^
*S. granarius*	CP1a	1.231	2.951	46.001 (18.294–115.673)	0.724	0.923
	Cta	1.583	2.780	25.259 (12.676–50.331)	0.855	0.991
	Lda	2.370	2.330	13.383 (8.506–21.055)	0.834	0.990
	Pba	1.672	2.578	28.082 (14.424–54.672)	0.861	0.993
	Pca	2.750	1.676	16.170 (10.838–24.125)	0.957	1.000
	PcIa	2.640	1.572	19.876 (12.975–30.449)	0.559	0.964
	Pg	2.399	2.325	13.034 (8.327–20.402)	0.970	1.000
	Pma	1.315	2.882	40.768 (17.267–96.256)	0.879	0.992
	Pra	2.116	2.263	19.632 (11.714–32.902)	0.605	0.956
	Pta	2.874	1.226	20.577 (13.793–30.697	0.786	0.994
*S. oryzae*	CP1a	2.733	1.023	28.499 (18.070–44.949)	0.819	0.996
	Cta	1.737	2.526	25.145 (13.326–47.466)	0.548	0.922
	Lda	2.129	2.808	10.727 (6.487–17.737)	0.512	0.861
	Pba	2.592	1.359	25.358 (16.053–40.358)	0.576	0.970
	Pca	2.144	1.803	30.984 (17.770–54.024)	0.801	0.992
	PcIa	1.568	2.621	32.913 (16.019–67.624)	0.997	1.000
	Pg	3.050	1.042	19.847 (13.599–28.964)	0.776	0.994
	Pma	1.737	2.526	25.145 (13.326–47.466)	0.548	0.922
	Pra	2.215	2.554	12.718 (7.850–20.606)	0.942	0.999
	Pta	4.421	−0.936	21.998 (16.400–29.507)	0.740	0.996
*S. zeamais*	CP1a	0.741	3.297	197.304 (39.290–990.825)	0.765	0.916
	Cta	2.511	1.491	24.972 (15.662–39.816)	0.823	0.995
	Lda	1.594	3.035	16.974 (8.751–32.924)	0.747	0.963
	Pba	1.504	0.326	14.390 (7.159–28.925)	0.486	0.808
	Pca	3.205	0.898	19.045 (13.290–27.292)	0.892	0.999
	PcIa	1.474	3.263	15.075 (7.390–30.751)	0.366	0.713
	Pg	3.050	1.042	19.847 (13.599–28.964)	0.776	0.994
	Pma	1.737	2.526	25.145 (13.326–47.466)	0.548	0.922
	Pra	1.897	2.538	19.838 (11.212–35.101)	0.530	0.922
	Pta	1.345	3.041	28.558 (12.726–64.087)	0.759	0.965
*R. dominica*	CP1a	3.296	0.559	22.235 (15.416–32.070)	0.715	0.992
	Cta	3.299	0.760	19.257 (13.539–27.388)	0.607	0.981
	Lda	2.754	2.032	11.968 (8.057–17.777)	0.648	0.959
	Pba	2.702	1.339	22.617 (14.718–34.756)	0.731	0.990
	Pca	3.205	0.898	19.045 (13.290–27.292)	0.892	0.999
	PcIa	1.799	2.749	17.815 (9.838–32.261)	0.653	0.947
	Pg	2.528	1.953	16.041 (10.425–24.682)	0.960	1.000
	Pma	2.399	2.096	16.240 (10.331–25.527)	0.695	0.976
	Pra	2.368	2.101	16.749 (10.587–26.499)	0.852	0.994
	Pta	1.637	2.396	38.895 (19.000–79.622)	0.748	0.977
*T. granarium*	CP1a	2.355	1.892	20.834 (12.969–33.468)	0.469	0.933
	Cta	2.146	2.022	24.831 (14.390–41.308)	0.569	0.957
	Lda	2.227	2.791	9.812 (6.040–15.940)	0.886	0.991
	Pba	2.592	1.359	25.358 (16.053–40.058)	0.576	0.970
	Pca	2.207	2.251	17.609 (10.773–28.773)	0.741	0.981
	PcIa	1.249	3.798	9.164 (3.955–21.236)	0.937	0.993
	Pg	2.368	2.101	16.749 (10.587–26.499)	0.852	0.994
	Pma	2.197	2.796	10.077 (6.171–16.455)	0.770	0.967
	Pra	1.717	3.015	14.328 (7.749–26.491)	0.647	0.927
	Pta	2.739	1.447	19.812 (13.108–29.945)	0.757	0.990

CL: confidence limits, LT_50_ values are considered significantly different when 95% confidence limits fail to overlap, χ^2^: calculated value of chi-square, R^2^: goodness of fit.

**Table 3:** Two-way ANOVA results for main effects and interactions for adult mortality of stored product beetle pests exposed to EPF.

Source	df	*S. granarius*		*S. oryzae*		*S. zeamais*		*R. dominica*		*T. granarium*	
		F	*p*	F	*p*	F	*p*	F	*p*	F	*p*
Fungal isolate	30, 959	5.23	<0.001	6.48	<0.001	2.38	<0.001	8.38	<0.001	3.88	<0.001
Exposure time	2, 959	10.43	<0.001	12.38	<0.001	10.11	<0.001	30.12	<0.001	7.12	<0.001
Fungal isolate × Exposure time	60, 959	4.43	<0.001	3.83	<0.001	4.13	<0.001	7.84	<0.001	2.34	<0.001

df: Numerator and denominator degrees of freedom.

## Data Availability

Not applicable.
